# Association between excess weight and asthma in adolescents of Niterói, Rio de Janeiro, Brazil

**DOI:** 10.1186/1939-4551-8-S1-A28

**Published:** 2015-04-08

**Authors:** José Leonardo Sardenberg, Fabio Kuschnir, Antônio José Alves Da Cunha, Pauline Kale, Antônio José Leal Costa, Renata Kuschnir

**Affiliations:** 1 Policlínica Geral Do Rio De Janeiro - Faculdade Medicina De Petrópolis, Brazil; 2 Rio De Janeiro State University, Brazil; 3 Instituto De Puericultura e Pediatria Martagao Gesteira, Federal University of Rio De Janeiro, Brazil; 4 Universidade Federal Do Rio De Janeiro, Brazil

## Background

The objective of this study was to evaluate the association between excess weight (EW) and asthma in adolescents.

## Methods

Cross-sectional study with students ages 10-14 years of public schools of Niterói, Rio de Janeiro State (RJ). For asthma diagnosis was used the *International Study of Asthma and Allergies in Childhood*(ISAAC) questionnaire. The classification of nutritional status was obtained by the Z-scores of Body Mass Index (BMI) for age. It was considered overweight (OW) values ≥ Z-score> +1 and obesity Z-score> +2. Chi-square test, prevalence ratio and their respective confidence intervals of 95% were used to evaluate the association between the prevalence of asthma ("wheezing in the last 12 months") and nutritional categories of EW. Then, we used logistic regression to study these associations adjusted for socio-demographics of the sample. Differences between the BMI and the presence of asthma were analyzed by Student's t-test. The adopted level of significance was 5%.

## Results

Between June/ December 2010, were evaluated 464 adolescents (54% female). The prevalence of asthma was 10.9%. Regarding nutritional category, 29.1% had OW and 12.1% obesity. No significant associations were observed between asthma and EW, even when adjusted for age and sex. The mean BMI was higher among asthmatic boys (21.05±6,42 SD x 20.01±3,49 SD;*p=0,47*), while the opposite occurred among the asthmatic girls that had lower BMI than non-asthmatic ones (20.64±1,79 SD x 21.78±4,80 SD;*p=0,53*). However, no significant statistics regarding this parameter differences occurred.

## Conclusions

There were no associations between asthma and EW in the study sample. However due to the concomitant high prevalence of these NCDs in Niterói, RJ and the high load of this conditions on the Brazilian health system, future longitidunal studies in this area must be carried out in our country.

